# Hypomagnesemia In Patients OF Critical Care And Alcohol Withdrawal Syndrome: A Review

**DOI:** 10.1192/j.eurpsy.2022.1187

**Published:** 2022-09-01

**Authors:** M. Chowdhury, B. Ponieman

**Affiliations:** JAMAICA HOSPITAL MEDICAL CENTER, Psychiatry, NEW YORK, United States of America

## Abstract

**Introduction:**

Magnesium is one of the crucial electrolytes that plays a significant role in maintaining various cellular and metabolic processes. Studies demonstrate that Hypomagnesemia is evident in patients of critical care unit and alcohol withdrawal syndrome. Low Magnesium level is associated several dreadful complications as such higher mortality, cardiac arrythmias, septic shock, prolonged ICU stay, increased need for intubations and delayed weaning from ventilation etc. Prescribing Magnesium with cautious supervision might prevent these alarming sequels. Value to determine Hypomagnesemia regarding critical patients is extremely significant to determine timing for possible interventions.

**Objectives:**

To review the impact and significance of low serum Magnesium level on prognosis of patients with critical care unit and alcohol withdrawal syndrome.

**Methods:**

To evaluate our reseach topic, we search through “Pubmed” and “Google Scholar” database using key words “Hypomagnesemia”, “Critical care” and “Alcohol withdrawal syndrome”, articles popped up. We select 5 articles on the basis of internal and external validity.

**Results:**

Level of Magnesium determination is extremely crucial to steer proper management in ICU, CDU, and critically ill patients. Studies reflecting most of the patients in critical care and alcohol withdrawal syndrome suffer from Hypomagnesemia. Most recent studies demonstrate that a level below 0.75 mmol/L is considered Hypomagnesemia for total Mg and level below 0.42 mmol/L for ionized Mg.

**Conclusions:**

Hypomagnesemia is associated with dire consequences and fatal outcomes for critical patients in terms of mortality,prolonged ICU stay,septic shock as well as need for mechanical ventilation.Supplementing Mg with careful monitoring could prevent lethal aftermath while treating patients of AWS and critical care.

**Disclosure:**

No significant relationships.

